# Editorial: Reviews in pathology of infectious diseases, volume II

**DOI:** 10.3389/fvets.2026.1794051

**Published:** 2026-02-11

**Authors:** Francisco J. Pallares, Jaime Gomez-Laguna, Francisco J. Salguero

**Affiliations:** 1 Department of Comparative Pathology, School of Veterinary Medicine, University of Córdoba, Córdoba, Spain; 2 Department of Pathology, School of Veterinary Science, University of Surrey, Guildford, United Kingdom; 3 Department of Pathology, United Kingdom Health Security Agency (UKHSA-Porton Down), London, United Kingdom

**Keywords:** editorial, infectious disease, pathology, review, virus

Infectious diseases in humans and animals play a very significant role in Global Health, with many zoonotic diseases being at the top priority for the international institutions, like WHO or WOAH for control and eradication. Currently, viral disease like highly pathogenic avian influenza (H5N1), expanding the host range (1), outbreaks of Mpox (2), and Marburg (3) virus infection in Africa, with some cases of the former in Europe, or recurrent outbreaks of Henipavirus infections in Asia (4), are of high importance for the public after having suffered a devastating pandemic like COVID-19 very recently.

When we decided to launch this Research Topic with Frontiers, we wanted to emphasize the importance of infectious diseases in animals, and zoonotic diseases in particular, and this commitment has been endorsed by the high number and quality of contributions received, which has prompted us to expand the contributions with a new volume. The present Research Topic, as in Volume I, continues its focus on the pathology of infectious diseases affecting animals and humans, emphasizing the value of animal models of disease to combat them.

Ruengket et al. described a proteomics and bioinformatics approach to profile serum proteins in wild stump-tailed macaques seropositive to Zika virus. They constructed a serum proteomic profile with 52 proteins associated with the inhibition of the apoptosis pathway, intracellular resource competition with the virus, neurological damage due to the virus and the host immune and defense responses.

Ruedas-Torres et al. described the histopathological lesions observed in the respiratory and nervous systems after intranasal and intraperitoneal inoculation with Nipah virus in a golden Syrian hamster model, using a combination of histopathological techniques including immunohistochemistry, *in situ* hybridization, and multiplex immunofluroscence. Their results helped to characterize this animal model of infection, that is currently used in preclinical testing of antiviral and vaccine strategies, and that could be also applied to studies of infections with other henipaviruses. Moreover, they have developed algorithms to characterize cell interactions in multiplex IF stained slides, what can be applied to multiple studies.

Zhang et al. described the pathological and gene expression changes after inoculation of Goose parvovirus in goslings. This virus induces high mortality and causes growth retardation and dwarfism in infected goslings. This study provide valuable information about the pathogenicity of the virus that could be used to mitigate its impact and improve the productivity of the waterfowl industry.

Sanchez-Rojas et al. described eight fatal cases of yellow fever in four different genera of captive non-human primates (*Cebus albitrons, Ateles fusciceps, Lagothrix lagotricha*, and *Aotus* spp.) housed at wildlife centers or found nearby. This is the first evidence of natural infection in *C. albitrons, A. fusciceps*, and *L. lagotricha* under managed care conditions what must be considered for vector control, biosafety measures and One Health-based interventions to prevent spillover and to be prepared for future outbreaks.

Akhtar et al. reviewed current insights into pathogenesis, clinical impact, and advances in treatment and vaccine development for feline immunodeficiency virus. The paper provides comprehensive and up-to-date summary of current knowledge and highlight those aspects that require further research to improve treatment and prevention of the virus infection.

Tutu et al. reviewed recent advances and future perspectives of the most common oral malignancy in cats, feline oral squamous cell carcinoma. The paper recovers the recent progress in understanding etiology, pathology, and therapeutic strategies, including ongoing challenges and future directions in the management of the process.

Gómez-Osorio et al. reviewed the disease caused in pig by *Lawsonia intracellularis*, porcine proliferative enteropathy, an economically significant health concern in global pig industry. The review includes current research on prevention and management strategies, and genetic resistance trends in swine, and discusses the implementation of biosecurity measures, cost-effectiveness, economic implications, and future perspectives of these strategies.

Kardoudi et al. describes the evaluation of clinical, analytical, and genotyping performance of the Hex L1 PCR combined with high-resolution melting curve analysis for investigating recent fowl adenovirus outbreaks in Morocco, including cases of inclusion body hepatitis and adenoviral gizzard erosion. This method provides a fast, sensitive, and reliable alternative for fowl adenovirus detection and genotyping, enabling universal detection, quantification, and genotyping in a single step.

Yeritsyan et al. reviewed 19 studies addressing both human and animal (cattle, small ruminants and swine) brucellosis in Armenia in order to better understand the country's epidemic situation. The manuscript summarizes the state of the start and identifies critical gaps in research and control efforts, offering insight that can support the need for a comprehensive, long-term national surveillance and control strategy.

Larenas-Muñoz et al. describes the evaluation of the effect of a prototype porcine circovirus type 2 vaccine on viral load and associated lesions compared with a commercial vaccine and a placebo control group. The control group showed more numerous and severe lesions compared to vaccinated groups, being the lesions milder in the commercial vaccine group than in the prototype vaccine group although no differences in the viral load were observed. Results obtained indicated the necessity of further development and optimization of the prototype formulation to enhance the protection against this important disease in the swine industry.
